# Total flavonoids of *Bidens bipinnata L.* ameliorate experimental adjuvant-induced arthritis through induction of synovial apoptosis

**DOI:** 10.1186/s12906-015-0962-3

**Published:** 2015-12-15

**Authors:** Ai-Zong Shen, Xia Li, Wei Hu, Fei-Hu Chen

**Affiliations:** Anhui Provincial Hospital Affiliated to Anhui Medical University, No. 17 Lujiang Road, Hefei, 230001 China; The third Hospital Affiliated to Nantong University, Wuxi, Jiangsu 214041 China; College of Pharmacy, Anhui Medical University, Hefei, 230032 Anhui People’s Republic of China

**Keywords:** Rheumatoid arthritis, Bidens bipinnata, Synovial membrane, Apoptosis

## Abstract

**Background:**

Bidens bipinnata are widely distributed in China, which have been widely used as a traditional Chinese medicine. The aim of this study was to examine the effect of total avonoids of Bidens pilosa L. (TFB) on adjuvant arthritis (AA) and its possible mechanisms.

**Methods:**

The macroscopic scoring of paw edema, secondary paw swelling, and polyarthritis index were measured. Histological examination of the joints and the serum concentrations of IL-6, IL-1beta, and TNF-alpha were examined. Apoptosis in synovial tissue was detected. The expression of Caspase 3 cleavage, serves as a marker undergoing apoptosis, was confirmed by Western blot.

**Results:**

TFB attenuated the severity of arthritis on paw edema, hind paw volume, and polyarthritis index of AA rats, improved the histological status in AA rats as well. TFB can inhibit the production of IL-6, IL-1beta, and TNF-alpha from serum. Clear DNA ladder formation was observed in DNA extraction of synovium from TFB treated AA rats. The number of apoptosis was increased with TFB treatment in TUNEL assay. TFB treatment on AA rats significantly increased the expression of Caspase 3 in synovium.

**Conclusions:**

Our data suggest that TFB has a signicant anti-arthritic effect in AA through the induction of apoptosis in synovial.

## Background

Rheumatoid arthritis (RA) is a chronic, systemic inflammatory disorder characterized by polyarthritis with progressive joint damage and disability, immunologic abnormalities, systemic inflammation, increased comorbidity, and premature mortality [1, 2]. It causes premature mortality, disability, and reduced quality of life in “developed” and “developing” countries [3]. The exact etiology of RA is not known, but infectious agents, oral contraceptives, smoking and genetic factors are believed to be responsible for the development of RA [4, 5].

Current therapeutic strategies in RA are focused on alleviation of the inflammatory reaction, joint pain and damage. Treatments include non-steroidal anti-inflammatory drugs (NSAIDs), glucocorticoids, specific inhibitors of pro-inflammatory mediators, non-biologic disease-modifying anti-rheumatic drugs (DMARDs) and biologic DMARDs [6]. However, most of these agents produce notable adverse effects, and searching for new agents with safe and effective therapy of RA has become essential [7–9].

Apoptosis is considered to be one of the mechanisms regulating autoimmune diseases such as RA [10–12]. It is well known that increased proliferation and insufficient apoptosis might contribute to the expansion of fibroblast-like synoviocytes (FLSs) in the pathogenesis of RA [13, 14]. Activated FLSs result in: synovium growth along with angiogenesis in articular cartilage; invasion into adjacent bone; stimulation of production of pro-inflammatory cytokines; destruction of cartilage and bone [15]. It has been shown that the induction of apoptosis in the synovium might be useful for the treatment of RA [16, 17].

The genus *Bidens* (Asteraceae), a medicinal plant used worldwide, has been traditionally used for the treatment hepatitis, inflammation, malaria, hypertension, diabetes, peptic ulcer, snake bite and smallpox [18, 19]. *Bidens bipinnata* L., commonly known as po-po-zhen (pinyin, China) in China, has also been applied in the treatment of jaundice, rheumatism, laryngitis, headache and digestive disorders [19, 20]. Pharmacological studies have shown that the extract of *Bidens pilosa* L. possess a wide range of biological activities, including antimalarial [21], antidiabetic [22], antioxidative [23], anticancer [24], anti-inflammatory [25], and immunomodulatory activity [26, 27]. Moreover, our previous study demonstrated that the total extracted flavonoids from *Bidens bipinnata* L. (TFB) had anti-inflammatory and antioxidant activity in acute liver injury [18] and liver fibrosis [19]. Futhermore, TFB is reported to inhibit the growth of mouse cervical carcinoma in U14 cells [28]. TFB has attracted much interest due to its proven pharmacologic safety and its many anti-inflammatory activities through inhibiting the production of pro-inflammatory cytokines such as interleukin (IL)-8, tumour necrosis factor (TNF)-a and nitric oxide (NO) levels in human umbilical vein endothelial cells [29]. RA is an inflammatory condition that affects joints primarily leading to pain and stiffness and difficulty in movements [30]. However, it has not been determined whether TFB is useful for inhibiting inflammation in RA. The resent study was designed to evaluate the therapeutic effect of TFB on RA in a rat model of adjuvant-induced arthritis (AA) and its possible mechanism of action.

## Methods

### Animals

Male Sprague–Dawley (SD) rats (180–200 g) were purchased from the Shanghai BK Experimental Animal Center (Shanghai, China). Rats were housed at 21 ± 2 °C and given free access to standard laboratory rat food pellets and water. Animals were distributed randomly to each treatment group of eight rats. Group size was determined as the minimum number of rats for valid statistical analyses. Animals were housed in a specific pathogen-free environment with free access to drinking water that was supplemented with gentamicin sulfate. Experiments were conducted according to the guidelines of the Institutional Animal Care and Use Committee of the Chinese Association for Laboratory Animal Sciences.

### Ethics statement

This study was carried out in strict accordance with the recommendations in the guidelines of the Institutional Animal Care and Use Committee of the Chinese Association for Laboratory Animal Sciences (approval number 2014AH-08-01). All experimental procedures involving animals were conducted as per institutional animal care guidelines of Nantong University and were approved ethically by the Jiangsu administration committee for experimental animals, China. All surgery was performed under diethyl ether, and all efforts were made to minimize suffering.

### Preparation of TFB

The dried aerial parts of Bidens bipinnata L. were collected from a local market and cut into small pieces. A voucher specimen (Chen 6128333) was deposited in the College of Pharmacy, Anhui Medical University. Professor Fan-Rong Wu confirmed the identity of each crude herb. The dried parts of Bidens pilosa L were extracted three times with 80 % ethanol at a tempareture of 80 °C for 2 h and filtered. The filtrates were concentrated in vacuo to give a syrup, followed by suspension water. Then, it was dissolved in water and filtered. The liquid was collected and passed through a column packed with HPD-100 resin. Elution was done using distilled water, 30 % ethanol, 50 % ethanol and 95 % ethanol. The 30 % ethanol-eluted solution was concentrated in vacuo to give TFB from the dried parts of Bidens bipinnata L.

### HPLC analysis

The preparation of the individual flavonoids were analyzed with the HP 1200 Agilent system (Agilent Technologies, Palo Alto, CA, USA) equipped with a dual pump, an auto-sampler and a PDA detector. Briefly, flavonoids were analyzed with an Agilent 1200 liquid chromatographic system (Agilent Technologies, Santa Clara, CA, USA) with an analytical column (250 mm × 4.6 mm I.D., SHIMADZU, Tokyo, Japan). The mobile phase consisted of 100 % acetonitrile (A) and water containing 0.5 % acetic acid (B) at a flow rate of 0.8 mL/min. The column temperature was set at 25 °C. The UV diode array detector was set at 280 nm and sample injection volume was 5 μl. Several standard compounds including hyperoside, rutin, maritimetin, quercetin, okanin, isookanin, protocatechuic acid, protocatechuic aldehyde, catechin, isoquercitrin, 7-O-(4”,6”-diacetyl)-b-d-glucopyranoside, (Z)-6-O-(3,6-di-O-acetyl-d-glucopyranosyl)-6,7,3’,4’-tetrahydroxyaurone, astragalin, and 2’,4’,6’-trimethoxy-4-O-d-glucopyranosyl-dihydrochalcone were run for comparative detection and were optimized. The calibration curves were defined for each compound in the range of sample quantity 0.02–0.5 μg. All samples were assayed in triplicate.

### Acute oral toxicity study

Healthy male Sprague–Dawley rats were subjected to acute toxicity studies as per Organization for Economic Co-operation and Development (OECD). The animals were fasted overnight and divided into group of 6 animals. The doses of 10, 50, 100, 150, 200, and 250 (mg/kg) were administered orally once a day for 14 days. Rats were weighed on days 2, 6, 10, and 14. Total food intake was measured as the weight of food consumed on days 2, 6, 10, and 14. Clinical signs and conditions associated with convulsion, ataxia, diarrhea, and impending death of animals were observed first 6 h and followed by 12 h after the administration and the animals in both control treated and TFB-treated groups were conducted daily during the period. On the 14rd day, rats were anesthetized and sacrificed. Tissues samples from the liver, spleen, lung, kidney, and brain were collected and embedded in paraffin for hematoxylin & eosin (H&E) staining.

### Creation and evaluation of the model of AA in rats

Creation of a model of AA was as described previously [31]. Rats were randomly divided by six groups (*N* = 8/group): Group a, normal control; Group b, AA; Group c, TFB 40 mg/kg + AA; Group d; TFB 80 mg/kg + AA; Group e; TFB 160 mg/kg + AA; and Group f, Glucoside tripterygium total (GTT, 10 mg/kg) + AA [32, 33]. Rats were immunized on day-0 by intradermal injection of a 0.1-mL aliquot of complete Freund’s adjuvant (CFA) containing 10 mg heat-inactivated bacillus Calmette-Guérin with paraffin oil into the left hind paw. The same volume of paraffin oil was injected into the left hind paw as a sham control. CFA-induced arthritis, assessed by the first signs of visible redness or swelling of the joints, was observed ≈ 10 days after each immunization in all groups (eight rats per group). Ten days to 24 days after CFA administration, rats receiving different doses of TFB (40, 80 and 160 mg/kg) were given TFB *via* gastric gavage. GTT; 10 mg/kg was used as a positive control. The volume of secondary paw swelling was measured with a plethysmometer (YLS-7A, Shandong, China). Secondary paw swelling due to edema was observed as an increase in the right hind paw by subtracting the paw volume at day-0 from that at days 10, 13, 17, 21 and 24, respectively. Moreover, the polyarthritis index of AA was measured as described previously [34].

### Histological processing and analyses of joints

After 28 days, rats were killed for histopathologic examinations. Thereafter, hind limbswere dissected, fixed with 10% phosphate-buffered formalin for 2 days, decalcified with 10% ethylenediamine tetraacetic acid for 7 days, and embedded in paraffin. Sections were prepared and stained with H&E and safranin-O staining for histologic processing and representative photographs taken.

### Measurement of serum concentrations of interleukin (IL)-6, IL-1β, and TNF-α

Animals were killed on day 28 after AA induction. Serum samples were collected and stored in −20 °C until analysis. Serum levels of IL-6, IL-1β and TNF-α were determined by enzyme-linked immunosorbent assay (ELISA) kits according to manufacturer instructions (Dakewe Biotic, Beijing, China).

### Detection of DNA ladders

The synovial tissues of the secondary paw swelling of rats were taken under the sterile condition. Fragmented DNA was isolated using a DNA Extraction kit (Sangon, Shanghai, China) according to manufacturer instructions. DNA extracts were electrophoresed on 1.5 % agarose gel containing ethidium bromide at 50 V/cm for 150 min. Ladder formation of oligonucleosomal DNA was detected under ultraviolet light.

### TUNEL assay

The TUNEL assay was carried out using an In Situ Apoptosis Detection kit (KeyGEN Biotech, Nanjing, China) according to manufacturer protocols. Sections were incubated with Labeling Safe Buffer containing TdT Enzyme and Biotin-conjugated dUTP at 37 °C for 60 min. Tissue sections were stained with FITC-conjugated extravidin and then counterstained with methyl green. To estimate apoptotic cell death, TUNEL-positive cell density was calculated as number of apoptotic cells per square millimeter of section.

### Western blot analysis

Aliquots (100 mg) of synovial tissues were homogenized and treated with RIPA Lysis Buffer. Protein concentration in lysates was measured using a BCA-200 Protein Assay kit (Pierce, Rockford, IL, USA). An equal amount (30 μg) of protein was separated by 12% SDS-PAGE and then transferred by electrophoresis. The primary antibody of caspase 3 (1:1000 dilution; Santa Cruz Biotechnology, Santa Cruz, CA, USA) was used for immunoblotting. Membranes were washed in Tris-buffered saline with Tween 20 (TBST), then incubated with secondary antibody (1:1000 dilution; Santa Cruz Biotechnology). The relative intensities of protein bands were quantified using densitometry (Gel Logic 2200; Carestream Health, Rochester, NY, USA).

### Statistical analyses

Data are the mean ± standard error of the mean. Statistical analyses were carried out using SPSS v11.0 (SPSS, license number:3605877, Chicago, IL, USA). The normality of distributions was tested. Statistical analyses were undertaken with one-way analysis of variance followed by a least significant difference *post hoc* test and unpaired Student’s t-test. *P* < 0.05 was considered significant.

## Results

### HPLC qualitative analysis

**Fig. 1:**
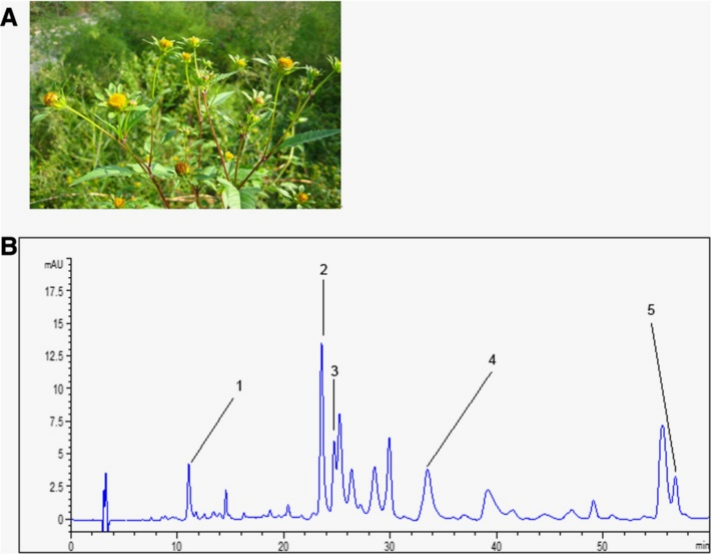
*Bidens bipinnata L.* picture and HPLC chromatograms of major flavonoids of *Bidens bipinnata L.* **a** *Bidens bipinnata L.* picture. **b** HPLC of flavonoids profile of *Bidens bipinnata L.*: (1) catechin; (2) hyperin; (3) isoquercitrin; (4) astragalin; (5) quercetin

The flavonoid composition of *Bidens bipinnata L.* was detected through HPLC analysis. HPLC analysis of *Bidens bipinnata L.* revealed the presence of chromatographic peaks consistent with the pattern showed by the standards such as catechin, hyperin, isoquercitrin, astragalin and quercetin. Quantitative HPLC analysis showed that hyperin (peak area, 17.855%) and astragalin (peak area, 13.648%) were the main flavonoids in *Bidens bipinnata L.* (Table 1; Fig. 1).

**Table 1:** Flavonoids profile of *Bidens bipinnata L.* through HPLC studies

Compound	Retention time (min)	Peak area(%)
Catechin	11.073	4.145 ± 0.671
Hyperin	23.553	17.855 ± 0.972
Isoquercitrin	24.710	2.674 ± 0.543
Astragalin	33.503	13.648 ± 0.754
Quercetin	56.800	3.818 ± 0.437

### Acute oral toxicity of TFB in rats

**Fig. 2:**
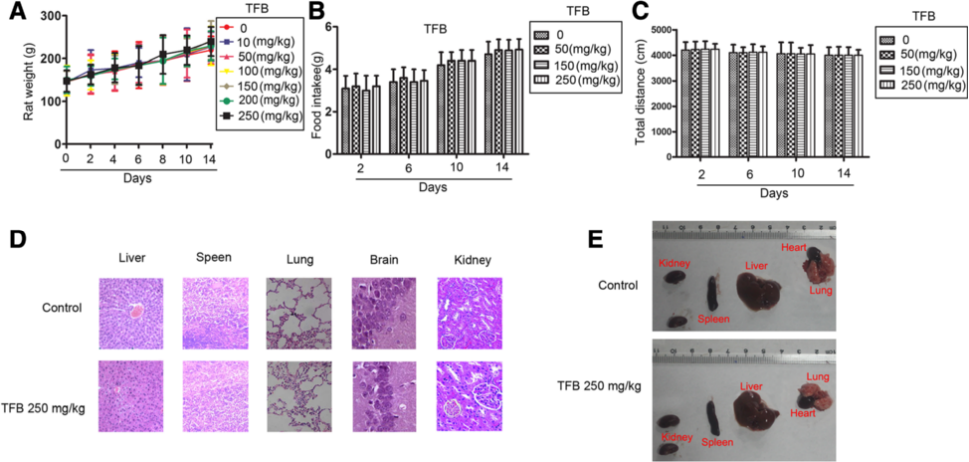
Toxicology evaluation of TFB. **a** and (**b**) Effect of TFB on body weight and food intake. Rats were treated with the indicated concentrations of TFB orally administered once a day for 14 days and body weights were measured with the indicated days. Rat total food intake was determined as the weight of food consumed during a 24-h period on days 2, 6, 10, and 14. **c** Locomotor activities of rats. The individual total running distance of rats was measured during a test period of 30 min on days 12, 6, 10, and 14. **d** HE staining of rat tissues after daily treatment with 0 or TFB at 250 mg/kg for 14 days. Scale bars indicate 50 μm. **e** Gross observation of systemic organs: liver, kidney, lung, heart and spleen from control and TFB treated rats

The safety of TFB was evaluated in rats after oral administration of 10, 50, 100, 150, 200, and 250 (mg/kg) once a day for 14 days. Body weight, food consumption, and locomotor activities were performed. As shown in Fig. 2a, b and c. No significant difference was found in body weight, mean daily food consumption, and locomotor activity. Moreover, No significant microscopic lesions were observed in the liver, spleen, lung, kidney, and brain histopathology in rats treated with TFB 250 (mg/kg) (Fig. 2d) as well as gross observation of systemic organs (Fig. 2e). The doses of 10–250 mg/kg of TFB did not exhibit any mortality or associated symptoms, such as convulsion, ataxia, and diarrhea (Table 2). These indicated that TFB had low toxicity profile.

**Table 2:** Clinical signs for control and treated groups

Observation	Control group		Test group	
	6 h	12 h	6 h	12 h
Convulsion	No	No	No	No
Ataxia	No	No	No	No
Diarrhea	No	No	No	No
Death	0/6*		0/6^a^	

^a^Number of dead rat/ number of rat used

### TFB inhibited paw volume and the polyarthritis index in AA rats

**Fig. 3:**
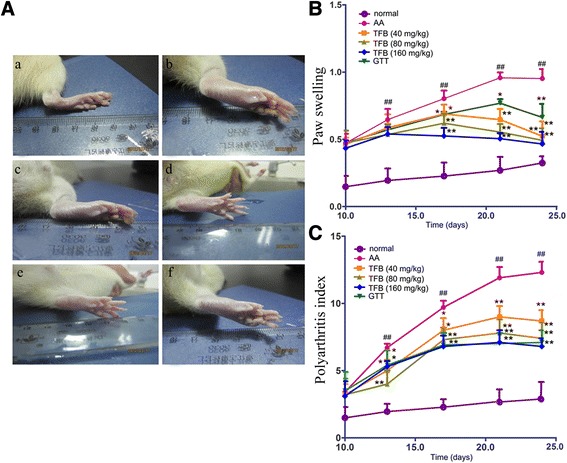
Pharmacological effects of TFB on CFA-induced arthritis in rats. **a** Photographic analyses of CFA-induced arthritis in rats. (a): Paw in a normal control; (b): Paw in AA control rat; (c): Paw in TFB (40 mg/kg)-treated AA rat; (d): Paw in TFB (80 mg/kg)-treated AA rat; (e): Paw in TFB (160 mg/kg)-treated AA rat; (f): Paw in GTT (10 mg/kg)-treated AA rat. **b** Effect of TFB on paw swelling (change in volume in mL) in rats with AA. **c** Effect of TFB on polyarthritis index in rats with AA. ^##^ *p* < 0.01 *vs* normal group; *p < 0.05, **p < 0.01 *vs* AA group

The preventive effect of TFB on AA rats was studied by evaluating the paw volume and the polyarthritis index of normal and drug-treated AA rats. TFB (40, 80 and 160 mg/kg, p.o.) was given to rats 10 days to 24 days after CFA administration. Macroscopic evaluation of paw edema in AA rats showed that paw edema to be induced in all experimental animals (Fig. 3a). Oral administration of TFB suppressed hindpaw edema. Inflammatory polyarthritis was induced in all immunized rats. The secondary inflammatory reaction appeared at ≈ day-10. Therefore, therapeutic administration of TFB (40, 80 and 160 mg/kg) was given at that time. CFA injection resulted in progressive swelling of the injected hind paw that increased over time up to day 24. Administration of TFB (40, 80 and 160 mg/kg) and GTT apparently decreased the paw volume and polyarthritis index in AA rats compared with arthritic control rats, and the efficacy was similar to that of GTT at 10 mg/kg (Fig. 3b and c).

### Effect of TFB on histologic changes in joints of AA rats

**Fig. 4:**
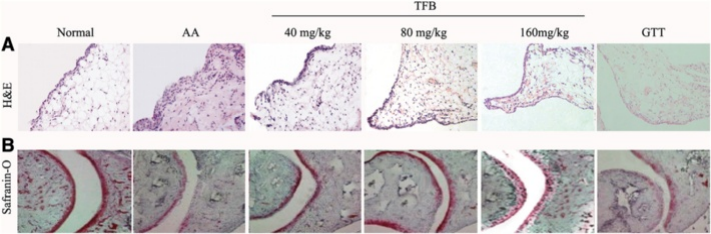
Histological assessment of arthritis in AA. Histological sections of ankle joints stained with (**a**) H&E (A, synovial inflammation, ×200) and (**b**) safranin-O (C, cartilage damage, ×200) were examined

Histologic analyses of experimental groups are illustrated in Fig. 4a. The characteristic features of synovial hyperplasia and inflammatory infiltration were observed in tissues from arthritic rats. After treatment with TFB for 8 days, synovial proliferation and infiltration of inflammatory cells in the synovium tissue were alleviated. Next, we examined the extent of cartilage damage in the joints of AA rats employing safranin-O staining (Fig. 4b). The joints of AA rats showed cartilage destruction, it was reduced significantly in the AA rats treated with TFB.

### Effect of TFB administration on concentrations of IL-1β, TNF-α, and IL-6 in the serum of AA rats

**Fig. 5:**
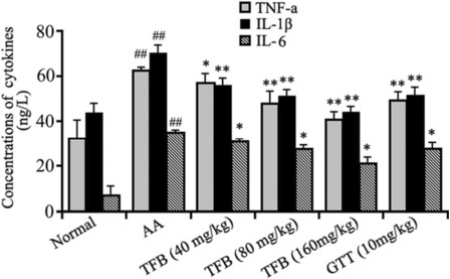
Effect of TFB administration on concentrations of IL-1β, TNF-α, and IL-6 in the sera of AA rats. Data are the mean ± SD, *n* = 8 per group. ##*p* < 0.01 *vs* normal group; *p < 0.05, **p < 0.01 *vs* AA group

At the end of the experiment, we measured the concentrations of IL-1β, TNF-α, and IL-6 in rat serum. These inflammatory cytokines are involved in RA. We examined the effect of TFB on the production of IL-1β, TNF-α, and IL-6 using ELISA. Levels of these inflammatory cytokines in serum induced by CFA were decreased significantly in TFB-treated groups compared with the model group (Fig. 5).

### Pro-apoptotic effects of TFB in vivo on synovial apoptosis in AA rats

**Fig. 6:**
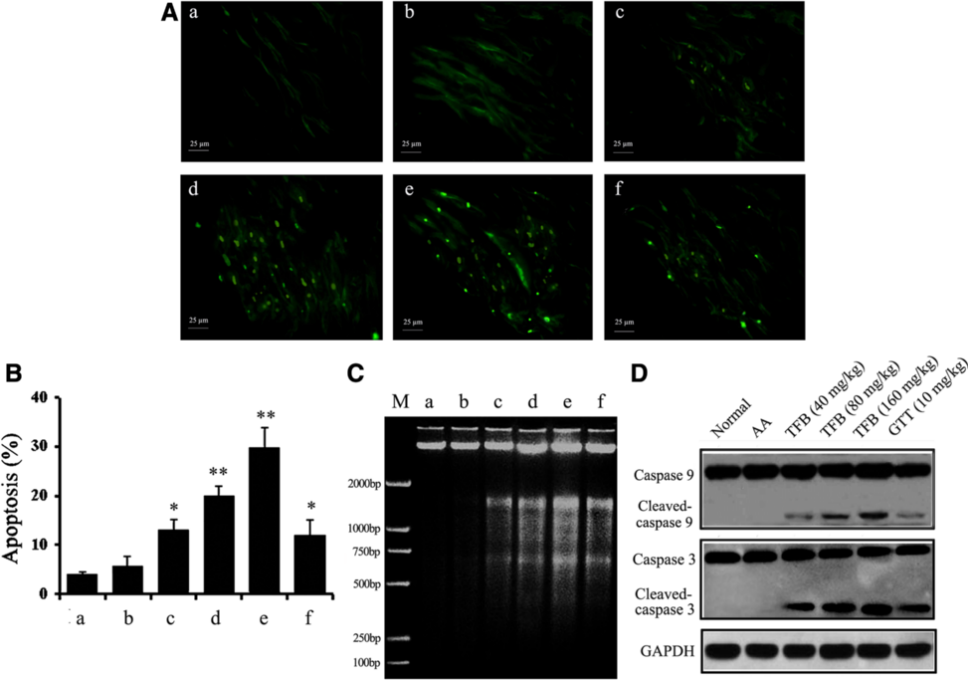
Effect of TFB on synovial apoptosis in the ankle joints of rats with AA. **a** Typical photographs of synovial-cell apoptosis from different groups. (*a*) Normal; (*b*) AA; (*c*) TFB (40 mg/kg); (*d*) TFB (80 mg/kg); (*e*) TFB (160 mg/kg); (*f*) GTT (10 mg/kg). **b** Quantitative evaluation of TUNEL-positive cells. Data are the mean ± SEM (*n* = 12). *p < 0.05, **p < 0.01 compared with the AA group. **c** Formation of DNA ladder patterns of synovial tissues in joints upon TFB treatment. (M) marker; (*a*) Normal; (*b*) AA; (*c*) TFB (40 mg/kg); (*d*) TFB (80 mg/kg); (*e*) TFB (160 mg/kg); (*f*) GTT (10 mg/kg). **d** Effects of TFB on expression of caspase-3 protein in synovial tissues from the ankle joints of AA rats

To ascertain if TFB induces synovial apoptosis, the TUNEL assay, detection of DNA ladders, and western blot analyses were carried out. Analyses of synovial-cell sections by the TUNEL assay clearly showed extensive apoptosis in AA rats treated with TFB (Fig. 6a). The number of TUNEL-positive cells was increased significantly in TFB (40, 80 and 160 mg/kg) treatment groups compared with the AA group (Fig. 6b). Agarose gel electrophoresis showed DNA ladders to be clearly embedded in TFB (40, 80 and 160 mg/kg) or GTT (10 mg/kg) (Fig. 6c). Caspase 3 (a typical “execution” caspase in apoptosis) was clearly activated by TFB because cleavage of caspase 3 was distinctly evident in TFB treatment groups (Fig. 6d).

## Discussion

RA is a common systemic autoimmune disease, which is characterized by pain and joint swelling, deformity, joint stiffness and serious functional damage. AA induced by FCA could act as an experimental model of RA in our study to demonstrate the effects of TFB on AA rats because of its similarity to human RA in both clinical and histopathologic features [35]. Interestingly, our results suggest that TFB significantly reduces AA severity and affects the contributory factors of arthritic inflammation (including production of inflammatory cytokines), which suggests that TFB is effective in the treatment of AA. The mechanisms of TFB treatment are related to increases in the apoptosis of synovial cells by regulation of activation of caspase 3.

It is well known that HPLC is gaining increasing importance for the analysis of plant extracts [36, 37]. The chemical analysis of total extracted flavonoids from *Bidens bipinnata* L. showed that hyperin and astragalin were the main flavonoids after following quantitative HPLC analysis. During the acute assessment of the TFB, there was no mortality and no other severe symptoms were observed up to the dose of 250 mg/kg in rat.

After FCA administration to rats, development of arthritis is observed by paw edema owing to soft-tissue swelling around ankle joints. Infiltration of polymorphonuclear leukocytes and lymphocytes from blood into joints is a key feature in human and experimental arthritis [38]. In our study, rats injected with CFA exhibited paw edema in arthritic rats, which might be concomitant with infiltration of leukocytes into joints [39]. An obvious reduction of paw swelling and the arthritis index observed in TFB-administered rats could be ascribed to the effects of TFB.

The inflamed joints of RA patients exhibit infiltration of inflammatory cells and hyperplasia, which is responsible for the degradation of cartilage and bone. According to the analyses of the histopathologic features of joints, we determined hyperplasia of the synovial membranes to be a key factor contributing to damage to joints and tissues in RA [40]. TFB could alleviate proliferation of the synovium, infiltration of inflammatory cells, and erosion of cartilage in arthritic rats and significantly relieve the swelling of ankles, which suggested that TFB possessed anti-inflammatory effects.

Pro-inflammatory mediators such as TNF-α, IL-1β and IL-6 have been reported to be responsible for the pathogenesis of RA based on their contribution to disease progression at different levels [41]. Therefore, they have been considered to be crucial cytokines in the pathogenesis of RA based on the marked clinical efficacy of anti-cytokine therapy [42, 43]. Previous study found that increased levels of IL-1β in the early phase of RA and in the synovial fluid of established RA patients [44, 45]. The importance of IL-1β in onset arthritis is further highlighted by reports of its ability to promote the differentiation of Th17 cells [46]. Recently, expression of TNF-α, IL-1β and IL-6 in AA rats has been examined by ELISA. We also demonstrated elevation of expression of TNF-α, IL-1β and IL-6 and the inhibitory effect from TFB in this model.

Research has confirmed that relatively insufficient apoptosis of synoviocytes in RA causes higher cellular proliferation and continuous synovial hyperplasia, leading to the destruction of cartilage and bone [16, 47]. Therefore, induction of synovial apoptosis is proposed to be one of the ways for treating RA by reducing synovial hyperplasia [16].

As indicated by previous studies, flavonoids from plant extracts could induce synoviocytes apoptosis and suppress proliferation of in AA rats [48] and induced apoptosis of fibroblast-like synovial cells in human RA [49]. Similarly, we found a pro-apoptotic effect of TFB on synovial apoptosis in rats with AA in vivo. In a representative image of DNA agarose gel electrophoresis, formation of apoptotic DNA ladders was found in DNA extracts of synovial tissues from TFB-treated groups. TFB increased the number of TUNEL-positive cells in the synovial tissues of AA rats. We also demonstrated that TFB treatment in rats with AA might induce synovial apoptosis *via* up-regulation of caspase-3 activity. From the study, extracts from from *Bidens bipinnata* L. showed significant total flavonoids contents. Therefore, further studies are needed to isolate pure compounds from the crude extract and to better understand the mechanism involved in the process of synovial apoptosis in RA.

## Conclusions

Our study showed that TFB has a remarkable protective action against FCA-induced AA in rats. TFB can significantly ameliorate disease severity as assessed by paw swelling, the polyarthritis index and histologic evaluation. The therapeutic effects of TFB against inflammation in AA rats were associated with its ability to induce synovial apoptosis in vivo through modulation of caspase-3 activation. Further work is needed to clarify the detailed mechanisms of action of TFB at the molecular level.
